# Physical Fitness and Health Profile of Adolescents Living in Amazonas

**DOI:** 10.1002/ajhb.70047

**Published:** 2025-05-05

**Authors:** Luciana Pereira Miranda, Duarte Henriques‐Neto, Francisney Izidio Leitão, Alex Barreto de Lima

**Affiliations:** ^1^ Physical Education Course Amazonas State University Amazonas Brazil; ^2^ Research Center for Sports Sciences, Health Sciences and Human Development University of Maia Maia Portugal

**Keywords:** adolescents, health, low‐income, physical fitness, public health

## Abstract

**Background:**

Physical fitness is an indicator of health in young populations. The aim of this study was to assess the health profile of adolescents living in the interior of the state of Amazonas, Brazil.

**Methods:**

The sample consisted of 1332 adolescents (701 girls) aged between 10 and 15 years. Body mass index, waist circumference, and waist‐to‐height ratio were the attributes used to assess anthropometric markers of body size. Physical fitness tests from the PROESP‐Br battery were used to assess muscular and cardiorespiratory fitness.

**Results:**

The results indicate high prevalences of overweight and obesity, with 25% for boys and 23.1% for girls. In addition, around 50% of the adolescents had insufficient levels of cardiorespiratory fitness and muscle strength. Boys showed a higher prevalence of a healthy risk profile in the different physical fitness tests in all municipalities.

**Conclusions:**

The results of this research are fundamental for the specific development of policies and strategies to promote health in young people with these social and geographical characteristics. The assessment of physical fitness in a school context can contribute to the development of public health policies for these populations, especially in communities with low economic resources.

## 1 Introduction

Physical fitness (PF) is a multidimensional construct, defined as a set of attributes related to health or physical performance. The level at which people possess these attributes can be assessed using specific tests (Caspersen et al. 1985).

The PF is recognized as an important indicator of health in childhood and adolescence (Marques et al. 2024; Ortega et al. 2008) and is associated with both present health and future well‐being (Henriksson et al. 2021; Joensuu et al. 2021; Raghuveer et al. 2020). The level of individual PF is influenced by biological or “nature” factors (such as genetic characteristics, gender, and maturity) and environmental or “nurture” factors (such as the level of habitual physical activity) (Bouchard et al. 2012; Yan et al. 2016).

The most common PF variables studied in the scientific literature are: localized muscular endurance, explosive strength, speed of movement, agility, coordination, balance, and cardiorespiratory fitness (Henriques‐Neto 2021). In addition, some authors include body mass index (BMI), waist circumference (WC), and waist‐to‐height ratio (WHtR) as components of PF (Marques et al. 2024; Yu et al. 2023). A low level of PF is a significant risk factor for cardiovascular disease (Timpka et al. 2014), type 2 diabetes (Zaccardi et al. 2015), hypertension (Sui et al. 2017), stroke (Högström et al. 2015), and mortality (Barry et al. 2014). The assessment of PF in adolescents is of great interest to public health worldwide, since chronic diseases in adulthood are often associated with the level of PF during adolescence (Dutra Sobral et al. 2022).

During the early stages of life, especially in childhood and adolescence, numerous cognitive, physical, physiological, and behavioral changes occur (Biddle et al. 2019). These changes, together with the adoption or not of active and healthy habits, are of great relevance not only in adulthood but also in old age (García‐Hermoso et al. 2022). The BMI assessment and analysis is fundamental for characterizing the level of health in young people (Kelly et al. 2024). However, it is important to recognize that BMI has significant limitations in distinguishing between fat and muscle mass (Petřeková et al. 2024). Another indicator of health through anthropometric measurements is WHtR. Studies indicate that measuring WHtR is an excellent indicator of metabolic health, specifically as an indicator of central obesity in younger populations (Eslami et al. 2023; Gray 2024). Moreover, assessing anthropometric markers of body size and PF helps to identify, control, and prevent diseases that affect population groups (Gaya et al. 2021) in the school context.

The reality of adolescents in the interior of the state of Amazonas is very different from that of large urban centers. In addition to geographical isolation, the lack of education, low human development index, as well as population organization (i.e., indigenous and riverside communities) can influence health indicators and the development of scientific studies (IBGE 2025b; Paim et al. 2011; Silva et al. 2005). Thus, based on specific geographical characteristics and the limited availability of data as health indicators, this investigation aimed to identify and analyze the PF and health profile of adolescents from the interior State of Amazonas, Brazil.

## 2 Methods

### 2.1 Participants and Study Design

**FIGURE 1:**
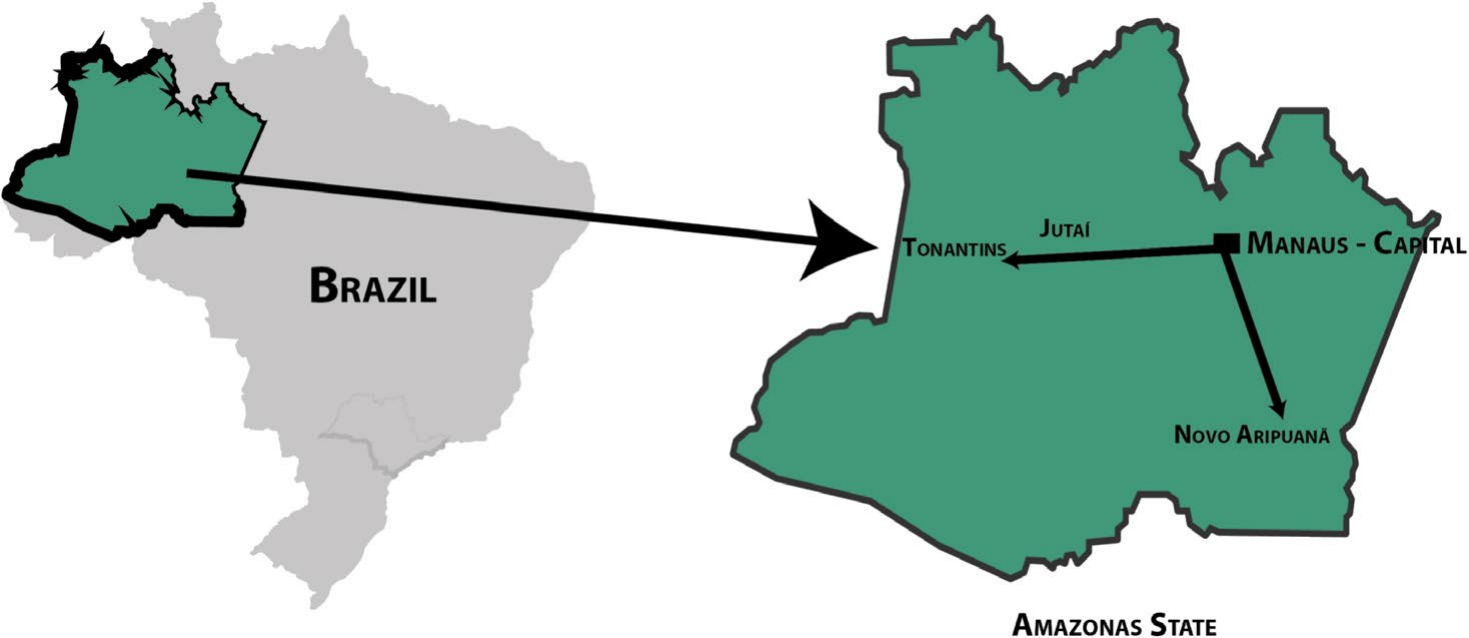
Geographical location of the municipalities.

A cross‐sectional study was carried out in the cities of Tonantins, Novo Aripuanã, and Jutaí, in the state of Amazonas (Figure 1). Tonantins (latitude 2°52′22″ S, longitude 67°48′07″ W) and Jutaí (latitude 2°44′49″ S, longitude 66°46′01″ W) are located in the upper Solimões region, 872 km and 985 km from the capital, respectively. Novo Aripuanã (latitude 5°08′00″ S, longitude 60°22′30″ W) is located in the micro‐region of the Madeira River, 1374 km from the capital of the state of Amazonas (Manaus) (IBGE 2025a). Access to these municipalities from Manaus and other Brazilian states is only possible by air or river. The majority of people living in the Amazon region use boats as their main means of transportation. These cities have predominantly rural characteristics, with economic activities centered almost exclusively on agriculture and fishing, with the Madeira River, the Solimões River, and their tributaries as the main means of subsistence and transportation. Migration from the riverside areas to the urban centers is largely due to the job opportunities and income generation provided by the recent urbanization process.

The sample consisted of 1332 children and adolescents aged between 10 and 15 years (631 boys; 701 girls) living in the municipalities of Tonantins, Novo Aripuanã, and Jutaí (Amazonas, Brazil). Participants were recruited through invitations from two public schools in each city. After explaining the study procedures to the participants and their guardians, both signed a consent form.

The socioeconomic level of the participants was assessed using a questionnaire from the Brazilian Association of Research Companies (ABEP 2003). This questionnaire stratifies the socioeconomic categories of the Brazilian population into five classes between A (those with greater purchasing power) and E (those with lower purchasing power) through a point classification. Thus, we identified the following social classes: Class A (45–100 points); Class B (29–44 points); Class C (17–28 points); Classes D and E (0–16 points).

The PF assessments were carried out in a multisport gymnasium by previously trained professionals. The authors of the study had no access to information that could identify individual participants during or after data collection.

Each participant took five PF tests from the PROESP‐Br Battery (Gaya et al. 2021) in two separate sessions with a 24‐h interval between each session. In the first session, anthropometric measurements (body mass, height and subcutaneous fat folds), flexibility, abdominal strength, and 20‐m run were initially assessed. The second session was designed to assess aerobic capacity, using the 6‐min run/walk test. Before carrying out the tests, the participants underwent a standardized 10‐min warm‐up conducted by the researchers.

To minimize the effects of circadian rhythm variability, all the tests were carried out in the same order and at the same time of day. The manual for PF tests, including procedures and protocols, is available on the PROESP‐Br website (https://lume.ufrgs.br/handle/10183/217804). The following were included in this study: (1) children and adolescents aged between 10.00 and 15.99 years, living in cities; (2) being independent in carrying out activities of daily living; (3) absence of contraindications for physical exertion (stroke, neurological diseases, unstable chronic conditions); and (4) absence of chest pain and/or angina pectoris.

Adolescents living in the rural areas of the municipalities were excluded due to the distance, as well as transportation to the urban area of the city, adolescents who were absent on the day of the assessments, or who had any difficulty getting around and carrying out the activities proposed by the project. All participants were informed about the aim of the project and written consent was obtained from their legal guardians before taking part in the research. This study was approved by the Ethics and Research Committee of the State University of Amazonas under protocol 5.621.338 and CAAE 56791822.8.0000.5016, in accordance with the Declaration of Helsinki (World Medical Association 2021) and Resolution 466/12 of the National Health Council (Ministério da Saúde, Brasil and Conselho Nacional de Saúde 2013).

### 2.2 Measurements

#### 2.2.1 Anthropometry and Body Composition

Body weight and height were obtained using a mechanical scale with a stadiometer attached (Welmy, São Paulo, Brazil), with the participants wearing shorts and t‐shirts, and all were barefoot. The BMI was calculated by dividing body mass (in kg) by height squared (in meters). All measurements followed the recommendations of the International Society for the Advancement of Kinanthropometry‐ISAK (Silva and Vieira 2020). WC was measured just above the lateral border of the ilium to an accuracy of 0.1 cm using a metal tape measure (Cescorf, Porto Alegre, Brazil), which aims to estimate abdominal adiposity (Xi et al. 2020). The WC was analyzed using the cutoff values from the FITescola battery (Henriques‐Neto et al. 2020; Jolliffe and Janssen 2007). After measuring height and WC, the WHtR was calculated, which is an indicator of central fat at any age (Ashwell and Hsieh 2005; Gaya et al. 2021). The WHtR of ≥ 0.5 was considered high risk (Hu and Staiano 2022; Lee et al. 2022).

#### 2.2.2 Maturity

Age at peak height velocity (APHV) was calculated using sex‐specific prediction equations from the research by Moore et al. (2015). The APHV has been defined as the age of maximum pubertal growth during adolescence and results from the difference between chronological age and the deviation from maturity.

In boys the predictive equation used was Maturity Offset = −7.999994 + [0.0036124 × (age × height)]: *R* ^2^ = 0.896; SEE = 0.542.

In girls, the predictive equation used was as follows: Maturity Offset = −7.709133 + [0.0042232 × (age × height)]: *R* ^2^ = 0.898; SEE = 0.528.

Maturity offset was calculated by the difference between chronological age and APHV age.

#### 2.2.3 PF Tests

The PROESP‐Br battery was used to assess PF (Gaya et al. 2021) and includes a set of tests that assess health‐related fitness in adolescents. The PF components assessed by the PROESP‐Br battery are cardiorespiratory fitness, muscular endurance, speed, flexibility, and body composition. Some specific tests from the PROESP battery used to assess each component of PF are described below. The specific cutoff values for each PF test were used to identify the health and risk profile.

#### 2.2.4 Sit and Reach Test

The sit and reach test was used to assess the flexibility of the lower back and the back of the thigh. A tape measure was placed on the ground and taped at the 38 cm mark. Participants were assessed barefoot, with their heels touching the tape at the 38 cm mark and 30 cm apart. The participants flexed their torso with both arms and legs extended, keeping their knees extended and their hands overlapping, palms facing down. Slowly, they leaned forward, extending their hands as far as possible, remaining in this position for as long as necessary to record the distance achieved. For this test, the best result of the two attempts was considered the final score (Gaya et al. 2021).

#### 2.2.5 The Abdominal Flexion Test

The 1‐min sit‐up test aims to assess the resistance strength of the muscles in the abdominal region. The student positioned himself in the supine position with his knees flexed at 45° and with his arms crossed over his chest. The evaluator, with both hands, holds the student's ankles, fixing them to the ground. At the “go” signal, the student began flexing the trunk until touching the elbows to the thighs, returning to the starting position. The student must perform the greatest number of complete repetitions in 1 min (Gaya et al. 2021).

#### 2.2.6 The 20 m Run

In the 20‐m run, time was recorded in seconds to assess the participants' acceleration and speed. Each participant positioned themselves behind the starting line, with one foot in front of the other in the starting position. At the sound of a whistle, the participant runs as quickly as possible to the finish line. The time was clocked and recorded. Each participant had the opportunity to perform two attempts, with a minimum rest interval of 3 min between them (Gaya et al. 2021).

#### 2.2.7 The 6‐Min Run

The 6‐min run/walk test aims to assess cardiorespiratory fitness, where the participant must complete as quickly as possible the greatest number of laps in 6 min, at a constant running pace. Walking at a fast pace is allowed, participants must maintain a constant cadence throughout the route, at the end of the time, and the total distance covered will be recorded. Afterwards, the participant must take a “return to calm” (Gaya et al. 2021).

### 2.3 Statistical Analysis

Statistical analyses were carried out using SPSS (version 28.0; IBM Corp). Descriptive statistics, including percentage, means, standard deviation (SD), and 95% confidence interval for mean (CI), were calculated for all outcome measures. Normality was assumed by the central limit theorem, given the size of the samples. Analysis of variance (ANOVA) was used to compare all the study variables between municipalities. The significance level adopted was *p* < 0.05.

## 3 Results

Table 1 shows the mean values for anthropometric variables, body composition, maturation, APHV, and each PF test. There were no statistically significant differences in body weight, height, BMI, abdominal flexion test, and 6‐min run/walk test between the three municipalities.

**TABLE 1:** Descriptive statistics for sex, body composition, and physical fitness tests by municipality.

Variables	*n* (%)/mean ± SD				*p*
	Total (1332)	Tonantins (515)	Jutaí (468)	Nova Aripuanã (349)	
Boys	631 (47.4)	282 (54.8)	129 (37)	220 (47)	—
Girls	701 (52.6)	238 (45.2)	202 (63)	248 (53)	
Socioeconomic status					
A	10 (8)	4 (0.8)	5 (1.1)	1 (0.3)	—
B	102 (7.7)	39 (7.6)	35 (7.5)	28 (0.8)	
C	253 (19)	100 (19.4)	92 (19.7)	61 (17.5)	
E	967 (72.6)	372 (72.2)	336 (71.8)	259 (74.2)	
Age (years)	12.8 ± 1.5	12.4 ± 1.6	13.2 ± 1.2	12.8 ± 1.6	< 0.001^a^, ^b^, ^c^
Weight (kg)	44.8 ± 11.9	44.6 ± 11.5	45.2 ± 12.0	44.7 ± 12.7	NS
Height (cm)	149.3 ± 11.1	149.6 ± 11.1	149.4 ± 11.0	148.8 ± 11.5	NS
BMI (kg/m^2^)	19.8 ± 3.4	19.6 ± 3.3	19.9 ± 3.4	19.8 ± 3.5	NS
Maturity offset	−0.3 ± 1.2	−0.6 ± 1.1	−0.06 ± 1.1	−0.2 ± 1.2	< 0.001^a^, ^b^
APHV	13.1 ± 1.2	13.09 ± 1.2	13.3 ± 1.07	13.02 ± 1.2	0.003^b^, ^c^
Waist circumference (cm)	63.6 ± 7.2	66.02 ± 8.9	61.2 ± 4.2	63.3 ± 6.3	< 0.001^a^, ^b^, ^c^
Waist‐to‐height ratio	0.4 ± 0.05	0.4 ± 0.06	0.4 ± 0.04	0.4 ± 0.05	NS
Sit and reach (cm)	40.5 ± 8.3	37.9 ± 9.7	43.03 ± 5.5	40.9 ± 8.3	NS
Abdominal flexion, *n*	21.6 ± 9.2	21.8 ± 9.2	21.5 ± 9.2	21.5 ± 9.3	NS
20 m run (s)	4.4 ± 0.2	4.4 ± 0.3	4.6 ± 0.2	4.5 ± 0.3	< 0.001^a^, ^b^, ^c^
6‐min run/walk test (m)	1021.04 ± 417.5	1021.5 ± 291.2	1009.8 ± 282.0	1035.4 ± 659.06	NS

Abbreviations: APHV, age of peak height velocity; BMI, body mass index; cm, centimeters; kg, kilograms; m, meters; *n*, numbers; NS, not significant; s, seconds; SD, standard deviation.

^a^ *p* ≤ 0.05, significant difference between Tonantins and Jutaí.

^b^ *p* ≤ 0.05, significant difference between Tonantins and Nova Aripuanã.

^c^ *p* ≤ 0.05, significant difference between Jutaí and Nova Aripuanã.

Table 2 shows the comparison of body composition characteristics and PF tests according to sex and municipality. Analyzing the sample data by gender based on the mean and 95% CI, boys have greater flexibility, are older, and reach APHV later. While girls, despite being younger according to chronological age, are more mature. The same trend can also be applied to WC, where the average for girls is higher than for boys. When analyzing the data by municipality, the sample shows significant differences in age, maturity, WC, flexibility, and speed between boys and girls from Tonantins, Jutaí, and Nova Aripuanã. The girls from Tonantins are significantly younger than those from Jutaí and Nova Aripuanã. Boys show a less marked difference, but it is still significant in Tonantins. The WC showed significant differences in both sexes, with adolescents in Jutaí showing lower values compared to the other municipalities.

**TABLE 2:** Comparison of body size and physical fitness tests by sex and municipalities.

Variables	All boys (631)	All girls (701)	Boys			Girls		
	Mean (95% CI)	Mean (95% CI)	Tonantins (282)	Jutaí (220)	Nova Aripuanã (129)	Tonantins (233)	Jutaí (248)	Nova Aripuanã (220)
Age (years)	13.1 (13.0 to 13.2)	12.5 (12.4–12.7)	12.9 (12.7 to 13.1)	13.3 (13.1 to 13.5)	13.3 (13.0 to 13.6)	11.8 (11.7 to 12.0)	13.2 (13.1–13.3)	12.5 (12.3–12.7)
Weight (kg)	44.7 (47.8 to 45.7)	45.0 (44.1–45.9)	44.1 (42.4 to 45.4)	45.5 (45.6 to 43.9)	44.6 (42.5 to 46.8)	45.3 (43.8 to 46.8)	44.9 (43.5–46.4)	44.8 (43.1–46.5)
Height (cm)	149.5 (148.6 to 150.4)	149.2 (148.4–150.0)	149.7 (148.4 to 151.1)	149.7 (148.5 to 151.2)	148.6 (146.6 to 150.6)	149.4 (148.0 to 150.8)	149.2 (147.8–150.5)	149.0 (147.4–150.5)
BMI (kg/m^2^)	19.7 (19.4 to 20.0)	19.9 (19.7–20.2)	19.4 (19.0 to 19.4)	20.0 (19.5 to 20.5)	19.8 (19.3 to 20.4)	20.0 (19.6 to 20.5)	19.9 (19.5–20.7)	19.8 (19.3–20.3)
Maturity offset	−0.9 (−0.99 to −0.8)	0.2 (0.1–0.3)	−1.005 (−1.1 to −0.9)	−0.8 (−0.9 to −0.70	−0.9 (−1.1 to −0.7)	−0.2 (−0.4 to −0.1)	0.6 (0.5–0.7)	0.2 (0.02–0.3)
APHV	14.0 (14.0 to 14.1)	12.3 (12.3–12.4)	13.9 (13.8 to 14.0)	14.1 (14.0 to 14.2)	14.2 (14.0 to 14.3)	12.1 (12.0 to 12.2)	12.6 (12.5–12.7)	12.4 (12.2–12.5)
Waist circumference (cm)	62.4 (61.8 to 62.9)	64.7 (64.2–65.3)	63.7 (62.7 to 64.7)	61.1 (60.5 to 61.7)	61.8 (60.6 to 63.1)	68.8 (67.7 to 70.0)	61.3 (60.8–61.8)	64.2 (63.4–65.0)
Waist‐to‐height ratio	0.4 (0.4 to 0.4)	0.4 (0.4–0.4)	0.4 (0.4 to 0.4)	0.4 (04 to 0.4)	0.4 (0.4 to 0.4)	0.5 (0.5 to 0.5)	0.4 (0.4–0.4)	0.4 (0.4–0.4)
Sit and reach (cm)	42.1 (41.4 to 42.7)	39.1 (38.5–39.7)	40.6 (39.5 to 41.6)	43.3 (42.5 to 44.1)	43.2 (41.5 to 44.9)	34.7 (33.5 to 35.9)	42.8 (42.2–43.4)	39.6 (38.7–40.6)
Abdominal flexion, *n*	21.7 (21.0 to 22.5)	21.6 (20.7–22.2)	22.1 (21.0 to 23.1)	21.2 (20.0 to 22.5)	21.9 (20.2 to 23.6)	21.5 (20.3 to 22.7)	21.8 (20.7–22.9)	21.3 (20.1–22.5)
20 m run (s)	4.5 (4.5 to 4.6)	4.4 (4.4–4.6)	4.5 (4.5 to 4.5)	4.6 (4.5 to 4.6)	4.6 (4.5 to 4.6)	4.3 (4.3 to 4.3)	4.6 (4.5–4.6)	4.4 (4.4–4.5)
6‐min run/walk test (m)	1019.9 (997.7 to 1042.3)	1007.8 (986.7–1028.8)	1039.1 (1004.5 to 1073.8)	1013.2 (975.9 to 1050.5)	989.6 (942.5 to 1036.7)	1000.2 (963.4 to 1037.0)	1006.8 (971.3–1042.3)	1016.8 (979.4–1054.3)

Abbreviations: APHV, age of peak height velocity; BMI, body mass index; cm, centimeters; kg, kilograms; m, meters; *n*, numbers; s, seconds; SD, standard deviation.

In the PF tests for boys, flexibility showed statistically significant differences between Tonantins/Novo Aripuanã and Tonantins/Jutaí. In speed running, only Tonantins and Jutaí showed statistically significant differences. Girls showed statistically significant differences between the municipalities in maturation, peak growth rate, WC, and WHtR.

In the PF tests for girls, flexibility and the 20 m speed test showed statistically significant differences between all the municipalities.

The percentage of boys and girls in the health risk zone and healthy zone for each PF and body composition test by the municipality is shown in Table 3. Considering the municipalities evaluated, BMI showed a prevalence (> 70%) of boys and girls classified as normal weight. In terms of WC, boys and girls were in the healthy zone with a prevalence of > 90%, except for girls in Tonantins who had 63.9% in the healthy zone. The WHtR in both sexes was in the healthy zone with a prevalence of > 85%, except for the girls from Tonantins who showed 73.4% in the healthy zone.

**TABLE 3:** Percentage (%) and 95% confidence interval of boys and girls in the health risk zone and healthy zone for each physical fitness and body composition test by municipality.

Variables	All (*n* = 1332)	Boys (*n* = 631)				Girls (*n* = 701)			
		Tonantins (*n* = 282)	Jutaí (*n* = 220)	Nova Aripuanã (*n* = 129)	Boys (*n* = 631)	Tonantins (*n* = 233)	Jutaí (*n* = 248)	Nova Aripuanã (*n* = 220)	Girls (*n* = 701)
BMI									
Normal	75.8 (73.5–78.1)	75.9 (70.9–80.9)	72.7 (66.8–78.6)	74.4 (66.9–81.9)	74.8 (71.1–77.9)	71.2 (65.4–77.1)	82.7 (78.0–87.4)	76.4 (70.8–82.0)	78.9 (73.7–80.0)
Overweight	16.6 (14.6–18.6)	18.1 (13.6–22.6)	19.1 (13.9–24.3)	17.1 (10.6–23.5)	18.2 (15.2–21.2)	19.3 (14.2–24.4)	12.5 (8.4–16.6)	13.6 (9.1–18.2)	15.1 (14.5–17.8)
Obesity	7.7 (6.2–10.22)	6.0 (3.3–8.8)	8.2 (4.6–11.8)	8.5 (3.7–13.4)	7.3 (5.3–9.3)	9.4 (5.7–13.2)	4.8 (2.2–7.5)	10 (6.0–14.0)	7.8 (6.0–10)
Waist circumference									
Health risk	8.7 (7.2–10.2)	2.8 (0.9–4.8)	—	—	1.3 (0.39–2.1)	36.1 (29.9–32.3)	1.6 (0.1–3.2)	9.1 (5.3–12.9)	15.4 (12.7–18.1)
Healthy	91.3 (89.7–92.8)	97.2 (95.2–99.1)	100 (100.0)	100 (100.0)	98.7 (97.7–99.6)	63.9 (58.8–70.1)	98.4 (96.8–99.9)	(90.9) (87.1–94.7)	84.6 (81.9–87.3)
Waist‐to‐height ratio									
Health risk	11.6 (9.8–13.3)	13.1 (9.2–17.1)	4.5 (1.8–7.3)	10.1 (66.8–78.6)	9.51 (7.2–11.8)	26.6 (20.9–32.3)	2.8 (0.8–4.9)	11.4 (7.2.–15.6)	13.4 (10.9–15.9)
Healthy	88.4 (86.7–90.2)	86.9 (82.9–90.8)	95.5 (92.7–98.2)	89.9 (84.7–95.1)	(90.5 (88.2–92.8)	73.4 (67.7–79.1)	97.2 (95.1–99.2)	88.6 (84.4–92.8)	86.6 (84.1–89.1)
Sit and reach									
Health risk	58.6 (56.0–61.3)	84.8 (80.6–88.9)	98.6 (97.1–100.0)	93.0 (88.6–97.4)	91.3 (89.1–93.5)	51.5 (45.1–54.9)	9.3 (5.6–12.9)	28.2 (22.2–34.1)	29.2 (25.9–32.6)
Healthy	41.4 (38.7–44.0)	15.2 (11.1–19.4)	1.4 (0.0–2.9)	7.0 (66.8–78.6)	8.7 (6.5–10.9)	48.5 (42.1–54.9)	90.7 (87.1–94.3)	71.8 (65.9–77.8)	70.8 (67.4–74.1)
Abdominal flexion									
Health risk	89.1 (87.4–90.8)	95.0 (92.5–97.6)	97.3 (95.1–99.4)	96.1 (92.8–99.5)	96.0 (94.5–97.6)	75.5 (70.0–81.1)	87.5 (83.4–91.6)	85.5 (80.8–91.1)	82.9 (80.1–85.7)
Healthy	10.9 (9.2–12.6)	5.0 (2.4–7.5)	2.7 (0.6–4.9)	3.9 (2.6–11.4)	3.7 (2.4–5.5)	24.5 (18.9–30.0)	12.5 (8.4–16.6)	14.5 (9.9–19.2)	17.1 (14.3–19.9)
20 m run									
Health risk	72.1 (69.7–74.5)	84.8 (80.6–88.9)	98.6 (97.1–100.0)	93.0 (88.6–97.4)	91.3 (89.1–93.4)	35.6 (29.5–41.8)	81.5 (76.6–86.3)	45.0 (38.4–51.6)	54.8 (51.1–58.5)
Healthy	27.9 (25.5–30.3)	15.2 (11.1–19.4)	1.4 (0.0–2.9)	7.0 (2.6–11.4)	8.74 (6.5–10.9)	64.4 (58.2–70.5)	18.5 (13.7–23.4)	55.0 (48.4–61.6)	45.2 (41.5–48.9)
6‐min run/walk test									
Health risk	41.9 (39.2–44.5)	44.7 (38.9–50.5)	51.4 (44.8–58.0)	50.4 (41.8–59.0)	48.2 (44.3–52.1)	31.3 (25.4–37.3)	42.7 (36.6–48.9)	34.1 (27.8–40.4)	36.2 (32.7–39.8)
Healthy	58.1 (55.5–60.8)	55.3 (49.5–61.1)	48.6 (42.0–55.2)	49.6 (41.0–58.2)	51.8 (47.9–55.7)	68.7 (62.7–74.6)	57.3 (51.1–63.4)	65.9 (56.7–72.2)	63.8 (60.2–67.3)

Abbreviation: BMI: body mass index.

In the sit and reach test, the boys were in the health risk zone with a prevalence (> 80%) along with the girls from Tonantins with 51.5%. The girls from Jutaí and Novo Aripuanã were in the healthy zone with a prevalence of > 70%. In abdominal flexion, boys and girls were in the health risk zone with a prevalence of > 75%. In the 20‐m run, the boys were in the health risk zone with a prevalence of > 85% along with the girls from Jutaí with 81.5%. The girls from Tonantins and Novo Aripuanã were in the healthy zone with a prevalence of > 55%. In the 6‐min run/walk, the girls were in the healthy zone with a prevalence (> 55%) along with the boys from Tonantins (55.3%). The boys from Jutaí and Novo Aripuanã were in the health risk zone with a prevalence of 50%.

The results of this study show that girls present a greater health risk than boys in the different variables analyzed. There were significant differences between the municipalities, specifically in BMI, abdominal strength, flexibility, and speed, especially between Tonantins and Jutaí. It is therefore understood that boys from Nova Aripuanã have better physical performance, especially in terms of strength and speed, when compared to Tonantins and Jutaí. In Jutaí, there are children with a higher health risk according to the average values of the flexibility and abdominal strength tests.

## 4 Discussion

This research aims to present the prevalence of the health profile of adolescents from three municipalities in the interior of the state of Amazonas, Brazil, through the evaluation of the different components of PF.

The PF is a biomarker of health for the population of all chronological ages. Assessing the different attributes of PF in the field using scientifically validated protocols makes it possible to identify health risks efficiently and at a low economic cost (Brazo‐Sayavera et al. 2024; Marques et al. 2021). Healthy Zone Profile (HZF) represents a lower risk of developing pathologies, particularly non‐communicable diseases such as obesity, cardiovascular diseases, diabetes mellitus, childhood sarcopenia, and mental illness at a younger age (Welk et al. 2015).

The results of this investigation show that the prevalence of overweight and obesity is between 24.1% and 27.3% in boys and 23.6%–38.7% in girls. This data is in line with the results of the study by Wang and Lim (2012), in which the regions of Europe and North America showed figures of between 20% and 40% prevalence of overweight and obese young people, while only areas such as Africa and Asia showed a prevalence of 10%–20%. Additionally, our results show a higher prevalence of overweight and obesity in all municipalities when compared to the prevalence of overweight and obesity in Brazilian adolescents (Guedes and Mello 2021; Zhang et al. 2024). The high prevalence of overweight and obesity in girls can be explained by their maturational state (Huh et al. 2011). However, this factor cannot be applied to the analysis of boys, since most of the boys are prepubescent, which requires a more careful analysis and the analysis of other factors (Aguirre et al. 2023) (i.e., social network, food patterns) that may have contributed to the results of this investigation, but was not assessed.

As the scientific community knows, BMI is an indicator of obesity that has generated some controversy among researchers, since it does not distinguish between the contribution of fat mass and lean mass to the final value (Gutin 2021). In this sense, WC and WHtR are more accurate solutions when it comes to assessing obesity in an epidemiological context. The WC and WHtR are measures related to abdominal obesity (Lee et al. 2022). These measures are the best indicators and predictors of the risk of non‐communicable diseases, such as cardiovascular disease, diabetes mellitus 2, and other metabolic diseases in young populations (Oliveira and Guedes 2018; Zimmet et al. 2007). Analysis of the WHtR results based on the health cutoff values established for health showed that the prevalence of health risk is between 4.5% and 13% in boys and 2.8%–26.6% in girls (Lee et al. 2022). The municipality of Tonantins has the highest numbers of adolescents in the health zone, 13% and 26.6% for boys and girls, respectively. Our results are high when compared with scientific studies in various areas of the world and indicate an increased risk factor for the development of metabolic diseases and hypertension in young populations (Lee et al. 2022; Pazin et al. 2020; Zong et al. 2023).

The ability to develop maximum force against a given external resistance is characterized by the component of PF called muscular fitness (Raghuveer et al. 2020). Muscle tissue plays a fundamental role in preventing muscle injuries, promoting functional independence, regulating body energy, and all causes of mortality (FitzGerald et al. 2004; Raghuveer et al. 2020). Muscle tissue is not only responsible for locomotion and the protection of internal organs, but it is also an endocrine organ responsible for producing myokines and other proteins that are fundamental to the global functioning of the human body (Argilés et al. 2016). High levels of muscular fitness in the 20 m speed test and sit‐ups or abdominal flexion test performance are indicators or predictors of low risk of developing pathologies related to bone tissue, metabolic syndrome, and mental illness (Henriques‐Neto et al. 2020; Steene‐Johannessen et al. 2009). In addition, high levels in the standing long jump and sit‐and‐reach tests are associated with high levels of academic performance at the ages studied (Gil‐Espinosa et al. 2020). In light of the aforementioned knowledge and the analysis of the results, the prevalence of health risk for boys of the sit‐to‐reach, abdominal flexion, and 20 m speed test is between 84.8% and 98.6%, 95%–97.1%, and 84.8%–98.6% respectively. Girls also showed a high prevalence of health risks, namely 9.3%–51.5% in the sit‐to‐reach, 75.5%–87.5% in the abdominal flexion, and 35%–81.5% in the 20 m speed test. The results for both sexes mean that the performance in these tests is mostly in the low percentiles concerning the health reference values for populations of these ages (Minatto et al. 2016; Tomkinson et al. 2018).

The prevalence of young people at health risk in the 6‐min run/walk test is between 44.7% and 51.4% in boys and 31.3%–42.7% in girls. These figures can be explained by the fact that high levels of obesity are associated with low levels of muscular fitness and cardiorespiratory fitness (Bagatini et al. 2023). With these high prevalence figures for adolescents at risk of health problems, we can say that around 50% of the population who took part in this investigation is at risk of developing cardiovascular pathologies, metabolic syndrome, mental illness, and low cognitive processes (Agostinis‐Sobrinho et al. 2022; Bernate et al. 2024; Raghuveer et al. 2020). It should be noted that adolescents with obesity and low levels of PF are at high risk of physical and mental comorbidities.

Thus, the analysis of this data is alarming concerning the health of adolescents living in the municipalities under study. However, the results may be general, and a potential intervention requires an individualized and contextualized analysis.

This investigation has limitations that must be taken into account. Firstly, the composition of the sample is not representative of the population of young people in the state of Amazonas‐Brazil, and for this fact, the interpretation of the data should be carried out with caution. Secondly, the cross‐sectional design of the investigation does not allow causality to be inferred from the results. Thirdly, the field evaluations, although carried out by physical education teachers, show errors when compared to PF tests with direct results (e.g., speed assessment with photoelectric cells and VO2_máx_ with gas analyzer). Despite the limitations identified, this research has several strengths that should be considered. As far as we know, this is the first inter‐municipal study in a geographical area with so many difficulties in terms of access and specialized material and human resources, with a sample of this size. Furthermore, this study is one of the few to provide strategies and results on the monitoring and prevalence of health profiles in young populations with low economic resources in this geographical area.

The PF is a biological and social multi‐construct, important for present and future health. (Lang et al. 2022). The results achieved in this investigation are fundamental for the specific development of policies and strategies to promote health in the young populations of these three municipalities. Government bodies (central and local), and health and education professionals need to understand the magnitude of health risk profiles in the young population, especially those living in low‐income situations and in geographical locations with difficult access to essential health goods.

## 5 Conclusions

In conclusion, this research presents new epidemiological results for the health profile of adolescents in the interior of the state of Amazonas, Brazil. Around one in four adolescents present health risks associated with overweight and obesity indicators, and around 50% are associated with low levels of cardiorespiratory fitness and muscle strength. This investigation shows that assessing PF in a school setting can help develop public health policies for these populations, especially in communities with low economic resources.

## Conflicts of Interest

The authors declare no conflicts of interest.

## Data Availability

The data that support the findings of this study are available from the corresponding author upon reasonable request.

## References

[ajhb70047-bib-0001] ABEP . 2003. “Critério de Classificação Econômica Brasil.” https://abep.org/wp‐content/uploads/2024/02/08_cceb_2003_em_vigor_em_2003_base_lse_2000.pdf.

[ajhb70047-bib-0002] Agostinis‐Sobrinho, C. , J. Kievišienė , A. Rauckienė‐Michaelsson , et al. 2022. “Cardiovascular Health Behavior and Cardiorespiratory Fitness in Adolescents: A Longitudinal Study.” European Journal of Pediatrics 181, no. 12: 4091–4099. 10.1007/s00431-022-04623-4. 36201018 PMC9540137

[ajhb70047-bib-0003] Aguirre, M. d. J. X. , F. C. Drumond Andrade , M. A. C. Aguirre , J. R. Justino , and B. L. L. Maciel . 2023. “Social Network, Food Patterns, Physical Activity and Associations With Overweight and Obesity in Adolescents From a School in Rural Brazil.” Nutrients 15, no. 15: 3305. 10.3390/nu15153305. 37571243 PMC10421155

[ajhb70047-bib-0004] Argilés, J. M. , N. Campos , J. M. Lopez‐Pedrosa , R. Rueda , and L. Rodriguez‐Mañas . 2016. “Skeletal Muscle Regulates Metabolism via Interorgan Crosstalk: Roles in Health and Disease.” Journal of the American Medical Directors Association 17, no. 9: 789–796. 10.1016/j.jamda.2016.04.019. 27324808

[ajhb70047-bib-0005] Ashwell, M. , and S. D. Hsieh . 2005. “Six Reasons Why the Waist‐to‐Height Ratio is a Rapid and Effective Global Indicator for Health Risks of Obesity and How Its Use Could Simplify the International Public Health Message on Obesity.” International Journal of Food Sciences and Nutrition 56, no. 5: 303–307. 16236591 10.1080/09637480500195066

[ajhb70047-bib-0006] Bagatini, N. C. , C. D. Feil Pinho , G. T. Leites , et al. 2023. “Effects of Cardiorespiratory Fitness and Body Mass Index on Cardiometabolic Risk Factors in Schoolchildren.” BMC Pediatrics 23, no. 1: 454. 10.1186/s12887-023-04266-w. 37689621 PMC10492303

[ajhb70047-bib-0007] Barry, V. W. , M. Baruth , M. W. Beets , J. L. Durstine , J. Liu , and S. N. Blair . 2014. “Fitness vs. Fatness on All‐Cause Mortality: A Meta‐Analysis.” Progress in Cardiovascular Diseases 56, no. 4: 382–390. 24438729 10.1016/j.pcad.2013.09.002

[ajhb70047-bib-0008] Bernate, J. , L. Rojas , and J. Mendoza . 2024. “Influencia de Las Habilidades físicas básicas en el Proceso Cognitivo: Una revisión sistemática (Influence of Basic Physical Skills on the Cognitive Process: A Systematic Review).” Retos 54: 84–93. 10.47197/retos.v54.101819.

[ajhb70047-bib-0009] Biddle, S. J. , S. Ciaccioni , G. Thomas , and I. Vergeer . 2019. “Physical Activity and Mental Health in Children and Adolescents: An Updated Review of Reviews and An Analysis of Causality.” Psychology of Sport and Exercise 42: 146–155.

[ajhb70047-bib-0010] Bouchard, C. , S. N. Blair , and W. L. Haskell . 2012. Physical Activity and Health. Human Kinetics.

[ajhb70047-bib-0011] Brazo‐Sayavera, J. , D. R. Silva , J. J. Lang , et al. 2024. “Physical Fitness Surveillance and Monitoring Systems Inventory for Children and Adolescents: A Scoping Review With a Global Perspective.” Sports Medicine 54, no. 7: 1755–1769. 10.1007/s40279-024-02038-9. 38710913 PMC11258155

[ajhb70047-bib-0012] Caspersen, C. J. , K. E. Powell , and G. M. Christenson . 1985. “Physical Activity, Exercise, and Physical Fitness: Definitions and Distinctions for Health‐Related Research.” Public Health Reports 100, no. 2: 126–131. 3920711 PMC1424733

[ajhb70047-bib-0013] Dutra Sobral, H. , J. Luiz Correia Júnior , M. B. Farias Duda , M. Pereira Gonçalves , M. Domingos Cardoso , and R. de Freitas Dias . 2022. “Perfil de Aptidão Física Relacionada à Saúde em Adolescentes Brasileiros: Revisão Integrativa da Literatura.” Id on Line. Revista de Psicologia 16, no. 61: 228–236.

[ajhb70047-bib-0014] Eslami, M. , F. Pourghazi , M. Khazdouz , et al. 2023. “Optimal Cut‐Off Value of Waist Circumference‐to‐Height Ratio to Predict Central Obesity in Children and Adolescents: A Systematic Review and Meta‐Analysis of Diagnostic Studies.” Frontiers in Nutrition 9: 985319. 10.3389/fnut.2022.985319. 36687719 PMC9846615

[ajhb70047-bib-0015] FitzGerald, S. J. , C. E. Barlow , J. B. Kampert , J. R. Morrow , A. W. Jackson , and S. N. Blair . 2004. “Muscular Fitness and all‐Cause Mortality: Prospective Observations.” Journal of Physical Activity and Health 1, no. 1: 7–18. 10.1123/jpah.1.1.7.

[ajhb70047-bib-0016] García‐Hermoso, A. , M. Izquierdo , and R. Ramírez‐Vélez . 2022. “Tracking of Physical Fitness Levels From Childhood and Adolescence to Adulthood: A Systematic Review and Meta‐Analysis.” Translational Pediatrics 11, no. 4: 474–486. 35558968 10.21037/tp-21-507 PMC9085944

[ajhb70047-bib-0017] Gaya, A. R. , A. C. A. Gaya , A. Pedretti , and J. B. Mello . 2021. Projeto Esporte Brasil, PROESP‐Br: Manual de Medidas, Testes e Avaliações. UFRGS.

[ajhb70047-bib-0018] Gil‐Espinosa, F. J. , P. Chillón , J. C. Fernández‐García , and C. Cadenas‐Sanchez . 2020. “Association of Physical Fitness With Intelligence and Academic Achievement in Adolescents.” International Journal of Environmental Research and Public Health 17, no. 12: 4362. 10.3390/ijerph17124362. 32570741 PMC7344740

[ajhb70047-bib-0019] Gray, L. A. 2024. “Evidence for Central Obesity Risk‐Related Thresholds for Adolescents Aged 11 to 18 Years in England Using the LMS Method.” Obesity Research & Clinical Practice 18, no. 4: 249–254. 10.1016/j.orcp.2024.07.002. 39019689

[ajhb70047-bib-0020] Guedes, D. P. , and E. R. B. Mello . 2021. “Prevalence of Overweight and Obesity Among Brazilian Children and Adolescents: Systematic Review and Meta‐Analysis.” ABCS Health Sciences 46: e021301. 10.7322/abcshs.2019133.1398.

[ajhb70047-bib-0021] Gutin, I. 2021. “Body Mass Index is Just a Number: Conflating Riskiness and Unhealthiness in Discourse on Body Size.” Sociology of Health & Illness 43, no. 6: 1437–1453. 10.1111/1467-9566.13309. 34086365 PMC8363552

[ajhb70047-bib-0022] Henriksson, P. , E. J. Shiroma , H. Henriksson , et al. 2021. “Fit for Life? Low Cardiorespiratory Fitness in Adolescence is Associated With a Higher Burden of Future Disability.” British Journal of Sports Medicine 55, no. 3: 128–129. 32816793 10.1136/bjsports-2020-102605 PMC10207998

[ajhb70047-bib-0040] Henriques‐Neto, D. 2021. Physical Fitness: Identifiers of Sport Participation and Bone Health in Youth. Universidade de Lisboa.

[ajhb70047-bib-0025] Högström, G. , A. Nordström , M. Eriksson , and P. Nordström . 2015. “Risk Factors Assessed in Adolescence and the Later Risk of Stroke in Men: A 33‐Year Follow‐Up Study.” Cerebrovascular Diseases 39, no. 1: 63–71. 25547343 10.1159/000369960

[ajhb70047-bib-0063] Henriques‐Neto, D. , J. P. Magalhães , M. Hetherington‐Rauth , D. A. Santos , F. Baptista , and L. B. Sardinha . 2020. “Physical Fitness and Bone Health in Young Athletes and Nonathletes.” Sports Health: A Multidisciplinary Approach 12, no. 5: 441–448. 10.1177/1941738120931755 PMC7485020 32660392

[ajhb70047-bib-0026] Hu, K. , and A. E. Staiano . 2022. “Trends in Obesity Prevalence Among Children and Adolescents Aged 2 to 19 Years in the US From 2011 to 2020.” JAMA Pediatrics 176, no. 10: 1037–1039. 10.1001/jamapediatrics.2022.2052. 35877133 PMC9315946

[ajhb70047-bib-0027] Huh, D. , E. Stice , H. Shaw , and K. Boutelle . 2011. “Female Overweight and Obesity in Adolescence: Developmental Trends and Ethnic Differences in Prevalence, Incidence, and Remission.” Journal of Youth and Adolescence 41, no. 1: 76–85. 10.1007/s10964-011-9664-4. 21499888 PMC3413457

[ajhb70047-bib-0028] IBGE . 2025a. Amazonas – Cidades e Estados. Instituto Brasileiro de Geografia e Estatística Accessed March 25, 2025. https://www.ibge.gov.br/cidades‐e‐estados/am/.

[ajhb70047-bib-0029] IBGE . 2025b. “Cidades.” Accessed March 6, 2025. https://www.ibge.gov.br/en/cities‐and‐states/am/.html.

[ajhb70047-bib-0030] Joensuu, L. , U. M. Kujala , A. Kankaanpää , et al. 2021. “Physical Fitness Development in Relation to Changes in Body Composition and Physical Activity in Adolescence.” Scandinavian Journal of Medicine & Science in Sports 31, no. 2: 456–464. 33038034 10.1111/sms.13847

[ajhb70047-bib-0031] Jolliffe, C. J. , and I. Janssen . 2007. “Development of Age‐Specific Adolescent Metabolic Syndrome Criteria That Are Linked to the Adult Treatment Panel III and International Diabetes Federation Criteria.” Journal of the American College of Cardiology 49, no. 8: 891–898. 17320748 10.1016/j.jacc.2006.08.065

[ajhb70047-bib-0032] Kelly, A. S. , S. C. Armstrong , M. P. Michalsky , and C. K. Fox . 2024. “Obesity in Adolescents.” JAMA 332, no. 9: 738. 10.1001/jama.2024.11809. 39102244

[ajhb70047-bib-0033] Lang, J. J. , K. Zhang , C. Agostinis‐Sobrinho , et al. 2022. “Top 10 International Priorities for Physical Fitness Research and Surveillance Among Children and Adolescents: A Twin‐Panel Delphi Study.” Sports Medicine 53, no. 2: 549–564. 10.1007/s40279-022-01752-6. 36001291 PMC9399984

[ajhb70047-bib-0034] Lee, J. , S.‐C. Kang , O. Kwon , S.‐s. Hwang , J. S. Moon , and J. Kim . 2022. “Reference Values for Waist Circumference and Waist–Height Ratio in Korean Children and Adolescents.” Journal of Obesity & Metabolic Syndrome 31, no. 3: 263–271. 10.7570/jomes22033. 36070974 PMC9579477

[ajhb70047-bib-0035] Marques, A. , Y. Demetriou , S. Popovic , et al. 2024. “Healthy Fitness Zone Prevalence and Age‐Specific Fitness Profile of Young People in Seven European Countries in 2022: The EUFITMOS Project.” American Journal of Human Biology 36, no. 2: e23989. 37732555 10.1002/ajhb.23989

[ajhb70047-bib-0036] Marques, A. , D. Henriques‐Neto , M. Peralta , et al. 2021. “Field‐Based Health‐Related Physical Fitness Tests in Children and Adolescents: A Systematic Review.” Frontiers in Pediatrics 9: 640028. 10.3389/fped.2021.640028. 33748047 PMC7973114

[ajhb70047-bib-0037] Minatto, G. , E. L. Petroski , and D. A. S. Silva . 2016. “Health‐Related Physical Fitness in Brazilian Adolescents From a Small Town of German Colonization.” Revista Andaluza de Medicina del Deporte 9, no. 2: 67–74. 10.1016/j.ramd.2014.09.003.

[ajhb70047-bib-0038] Ministério da Saúde, Brasil , and Conselho Nacional de Saúde . 2013. “Resolução n° 466, de 12 de dezembro de 2012.” Diário Oficial da União 12: 59.

[ajhb70047-bib-0039] Moore, S. A. , H. A. McKay , H. Macdonald , et al. 2015. “Enhancing a Somatic Maturity Prediction Model.” Medicine & Science in Sports & Exercise 47, no. 8: 1755–1764. 25423445 10.1249/MSS.0000000000000588

[ajhb70047-bib-0041] Oliveira, R. G. , and D. P. Guedes . 2018. “Performance of Anthropometric Indicators as Predictors of Metabolic Syndrome in Brazilian Adolescents.” BMC Pediatrics 18, no. 1: 33. 10.1186/s12887-018-1030-1. 29415673 PMC5804068

[ajhb70047-bib-0042] Ortega, F. B. , J. R. Ruiz , M. J. Castillo , and M. Sjöström . 2008. “Physical Fitness in Childhood and Adolescence: A Powerful Marker of Health.” International Journal of Obesity 32, no. 1: 1–11. 18043605 10.1038/sj.ijo.0803774

[ajhb70047-bib-0043] Paim, J. , C. Travassos , C. Almeida , L. Bahia , and J. Macinko . 2011. “The Brazilian Health System: History, Advances, and Challenges.” Lancet 377, no. 9779: 1778–1797. 10.1016/s0140-6736(11)60054-8. 21561655

[ajhb70047-bib-0044] Pazin, D. C. , T. L. da Luz Kaestner , M. Olandoski , et al. 2020. “Association Between Abdominal Waist Circumference and Blood Pressure in Brazilian Adolescents With Normal Body Mass Index.” Global Heart 15, no. 1: 27. 10.5334/gh.779. 32489800 PMC7218763

[ajhb70047-bib-0045] Petřeková, K. , N. Borzenko , M. Kovalová , and N. Gottfriedová . 2024. “Assessment of Body Mass Index, Body Composition, Physical Activity, and Dietary Preferences in University Students: A Pilot Study.” Obesities 4, no. 1: 35–44.

[ajhb70047-bib-0046] Raghuveer, G. , J. Hartz , D. R. Lubans , et al. 2020. “Cardiorespiratory Fitness in Youth: An Important Marker of Health: A Scientific Statement From the American Heart Association.” Circulation 142, no. 7: e101–e118. 32686505 10.1161/CIR.0000000000000866 PMC7524041

[ajhb70047-bib-0047] Silva, H. P. , D. E. Crews , and W. A. Neves . 2005. “Subsistence Patterns and Blood Pressure Variation in Two Rural Caboclo Communities of Marajó Island, Pará, Brazil.” American Journal of Human Biology 7, no. 4: 535–542. 10.1002/ajhb.1310070415. 28557098

[ajhb70047-bib-0048] Silva, V. S. , and M. F. S. Vieira . 2020. “International Society for the Advancement of Kinanthropometry (ISAK) Global: International Accreditation Scheme of the Competent Anthropometrist.” Revista Brasileira de Cineantropometria & Desempenho Humano 22: e70517.

[ajhb70047-bib-0049] Steene‐Johannessen, J. , S. A. Anderssen , E. Kolle , and L. B. Andersen . 2009. “Low Muscle Fitness is Associated With Metabolic Risk in Youth.” Medicine & Science in Sports & Exercise 41, no. 7: 1361–1367. 10.1249/MSS.0b013e31819aaae5. 19516166

[ajhb70047-bib-0050] Sui, X. , M. A. Sarzynski , D.‐C. Lee , and P. F. Kokkinos . 2017. “Impact of Changes in Cardiorespiratory Fitness on Hypertension, Dyslipidemia and Survival: An Overview of the Epidemiological Evidence.” Progress in Cardiovascular Diseases 60, no. 1: 56–66. 28274819 10.1016/j.pcad.2017.02.006

[ajhb70047-bib-0051] Timpka, S. , I. F. Petersson , C. Zhou , and M. Englund . 2014. “Muscle Strength in Adolescent Men and Risk of Cardiovascular Disease Events and Mortality in Middle Age: A Prospective Cohort Study.” BMC Medicine 12: 62. 24731728 10.1186/1741-7015-12-62 PMC4006633

[ajhb70047-bib-0052] Tomkinson, G. R. , K. D. Carver , F. Atkinson , et al. 2018. “European Normative Values for Physical Fitness in Children and Adolescents Aged 9–17 Years: Results From 2 779 165 Eurofit Performances Representing 30 Countries.” British Journal of Sports Medicine 52, no. 22: 1445–1456. 10.1136/bjsports-2017-098253. 29191931

[ajhb70047-bib-0053] Wang, Y. , and H. Lim . 2012. “The Global Childhood Obesity Epidemic and the Association Between Socio‐Economic Status and Childhood Obesity.” International Review of Psychiatry 24, no. 3: 176–188. 10.3109/09540261.2012.688195. 22724639 PMC4561623

[ajhb70047-bib-0054] Welk, G. J. , P. F. Saint‐Maurice , and T. Csányi . 2015. “Health‐Related Physical Fitness in Hungarian Youth: Age, Sex, and Regional Profiles.” Research Quarterly for Exercise and Sport 86, no. sup1: S45–S57. 10.1080/02701367.2015.1043231. 26054956

[ajhb70047-bib-0055] World Medical Association . 2021. Declaration of Helsinki: Medical Research Involving Human Subjects. World Medical Association Accessed July, 12.

[ajhb70047-bib-0056] Xi, B. , X. Zong , R. Kelishadi , et al. 2020. “International Waist Circumference Percentile Cutoffs for Central Obesity in Children and Adolescents Aged 6 to 18 Years.” Journal of Clinical Endocrinology & Metabolism 105, no. 4: e1569–e1583. 10.1210/clinem/dgz195. 31723976 PMC7059990

[ajhb70047-bib-0057] Yan, X. , I. Papadimitriou , R. Lidor , and N. Eynon . 2016. “Nature Versus Nurture in Determining Athletic Ability.” Medicine and Sport Science 61: 15–28. 27287074 10.1159/000445238

[ajhb70047-bib-0058] Yu, T. , Y. Jiang , J. Fan , et al. 2023. “Rapid Increases in BMI Waist to Height Ratio During Adolescence and Subsequent Neurobehavioral Deficits.” Obesity 31, no. 11: 2822–2833. 10.1002/oby.23881. 37735781

[ajhb70047-bib-0059] Zaccardi, F. , G. O'Donovan , D. R. Webb , et al. 2015. “Cardiorespiratory Fitness and Risk of Type 2 Diabetes Mellitus: A 23‐Year Cohort Study and a Meta‐Analysis of Prospective Studies.” Atherosclerosis 243, no. 1: 131–137. 26386209 10.1016/j.atherosclerosis.2015.09.016

[ajhb70047-bib-0060] Zhang, X. , J. Liu , Y. Ni , et al. 2024. “Global Prevalence of Overweight and Obesity in Children and Adolescents.” JAMA Pediatrics 178, no. 8: 800–813. 10.1001/jamapediatrics.2024.1576. 38856986 PMC11165417

[ajhb70047-bib-0061] Zimmet, P. , K. G. M. M. Alberti , F. Kaufman , et al. 2007. “The Metabolic Syndrome in Children and Adolescents? An IDF Consensus Report.” Pediatric Diabetes 8, no. 5: 299–306. 10.1111/j.1399-5448.2007.00271.x. 17850473

[ajhb70047-bib-0062] Zong, X. , R. Kelishadi , Y. M. Hong , et al. 2023. “Establishing International Optimal Cut‐Offs of Waist‐to‐Height Ratio for Predicting Cardiometabolic Risk in Children and Adolescents Aged 6–18 Years.” BMC Medicine 21, no. 1: 442. 10.1186/s12916-023-03169-y. 37968681 PMC10647138

